# An Updated Evaluation of the Dichotomous Link Between Creativity and Mental Health

**DOI:** 10.3389/fpsyt.2021.781961

**Published:** 2022-01-17

**Authors:** Rongjun Zhao, Zhiwen Tang, Fang Lu, Qiang Xing, Wangbing Shen

**Affiliations:** ^1^School of Education, Guangzhou University, Guangzhou, China; ^2^Zhongshan Institute, University of Electronic Science and Technology of China, Zhongshan, China; ^3^Jiangsu Provincial Key Constructive Laboratory for Big Data of Psychology and Cognitive Science, Yancheng Teachers College, Yancheng, China; ^4^Institute of Situational Education and School of Education, Nantong University, Nantong, China; ^5^School of Public Administration, Hohai University, Nanjing, China

**Keywords:** dual-pathway model, psychopathology, mental health, creativity, emotion regulation

## Abstract

The theory of the mad genius, a popular cultural fixture for centuries, has received widespread attention in the behavioral sciences. Focusing on a longstanding debate over whether creativity and mental health are positively or negatively correlated, this study first summarized recent relevant studies and meta-analyses and then provided an updated evaluation of this correlation by describing a new and useful perspective for considering the relationship between creativity and mental health. Here, a modified version of the dual-pathway model of creativity was developed to explain the seemingly paradoxical relationship between creativity and mental health. This model can greatly enrich the scientific understanding of the so-called mad genius controversy and further promote the scientific exploration of the link between creativity and mental health or psychopathology.

## Introduction

Mental health and creativity are the two critical elements driving the sustainable development of human society. With the continued spread of the COVID-19 pandemic around the world, there is an urgent need to address the deepening threats to individuals' mental health and creativity. According to the World Health Organization (1), mental illness, unlike genius, is not a rare phenomenon. In a recent report that surveyed U.S. adults at the end of June 2020, 31% of the respondents reported symptoms of anxiety or depression, 13% reported starting or increasing substance use, 26% reported stress-related symptoms, and 11% reported having had serious suicidal thoughts in the preceding 30 days. These numbers are almost double the rates estimated before the pandemic (2). The ongoing COVID-19 pandemic, an event rife with uncertainty and challenge, has led to a sharp rise in the demand for creativity often seen during such periods of unpredictability and change (3). Essentially, creativity can not only help people find meaning and significance during the pandemic by, for example, giving individuals enjoyment and pleasure but also help them feel an increased sense of purpose in a variety of ways, e.g., by producing better career narratives about their meaning-making at work (4). However, this is not the only reason behind the thirst for creativity during the epidemic; the search for creativity has also stemmed from its importance in scientific discovery and technological breakthroughs. Creativity generally involves the production of original and valuable ideas that can help scientists and medical professionals achieve innovative breakthroughs in epidemic management and vaccine development and therefore save more people. In this sense, examining the association between creativity and mental health is important [e.g., (5)].

Another important reason to examine this issue is the longstanding interest in the madness-creativity nexus or the mad genius hypothesis (6, 7), as illustrated by creative people who suffer or have suffered from serious mental disorders, such as Vincent van Gogh, making this nexus one of the oldest, most controversial and most frequently discussed issues in the domain of creativity (8, 9). In recent decades, many meaningful results have accumulated, including in journal articles [e.g., (8, 9)], chapters [e.g., (10)] and books [e.g., (11, 12)]. Overall, the association between creativity and mental health is an important issue, as partially illustrated by the emergence of the *Journal of Creativity in Mental Health*. Although the studies mentioned above have made important empirical and conceptual advancements, they are mostly fragmented and scattered and do not provide an integrated, accurate or coherent understanding of the topic (13). Given that several authors have already conducted systemic or scoping reviews, this study takes a different approach to update our understanding of the relationship between creativity and mental health. Specifically, the present study involves a state-of-the-art review, wherein a dichotomous approach is taken to integrate the potential positive and negative association between creativity and mental health.

This study is a narrative review that makes at least three contributions. First, it offers a state-of-the-art introduction on mental health and creativity beyond the so-called mad genius hypothesis. Second, this study attempts to profile and theoretically integrate the plausible but seemingly paradoxical association between creativity and mental health from a novel perspective. Third, the present review helps advance related studies on the association between creativity and psychopathology and on the relationship between creativity and mental health (well- and ill-being). Taken together, we provide many useful insights and helpful scaffolding knowledge about theoretical research and practices regarding creativity and/or mental health. The remainder of this paper is structured as follows. We first conceptualize creativity and mental health and then briefly review the findings in the paradox of these two constructs. Next, we critically evaluate previous studies on the association between creativity and mental health and present a novel theoretical account. Finally, the paper ends by describing the new state of the art and recommending directions for future research.

## The Paradox of Creativity and Mental Health

This section describes the three steps involved in our research. First, we conceptualize creativity and mental health. Then, we briefly review the negative association between creativity and mental health. Finally, we move to a detailed review of the literature on the positive association between creativity and mental health.

The first step in our analysis was conceptualizing creativity and mental health. Although defining creativity may be easy, establishing a consensual definition of creativity is not. Pursuing differing priorities and focuses, recent studies have provided useful insights into how to conceptualize creativity (14–16). According to one standard definition, creativity is the capacity to produce something new/novel and appropriate/useful within a certain sociocultural context (e.g., devices, ideas, or procedures); this capacity encompasses a creative personality/disposition and creative thinking and typically manifests in a variety of human activities, ranging from everyday life (e.g., environmental adaptation) to advanced technological industries [e.g., medical revolution; see (17)]. Importantly, individual maladaptation to a changing environment can easily result in mental illness and negative mental health trends [see (18)]. Generally, mental health is not a subjective status of the absence of disease but “a state of well-being in which the individual realizes his or her own abilities, can cope with the normal stresses of life, can work productively and fruitfully, and is able to make a contribution to his or her community” (19). This involves positive emotion and positive functioning, which individually represent emotional and psychological well-being (20).

A negative link between creativity and mental health has been speculated for centuries (21), and this idea is still widespread and deeply engrained in contemporary culture (22). Early evidence consisted of observations and examples from the lives of creative individuals (18). The often-cited biographical reports of luminaries, such as Sylvia Plath, as well as those of contemporary creatives, including Carrie Fisher and Amy Winehouse, all provide anecdotal support for the negative connection between creativity and mental health. Empirically, the frequently cited studies by Jamison (23), Andreasen (6), and Ludwig (12), which show a link between mental illness and creativity, have been criticized on the grounds that they involve small, highly specialized samples, use weak and inconsistent methodologies and strongly depend on subjective and anecdotal accounts (24). However, many recent strictly designed investigations have replicated the positive association between mental illness and creativity. Rybakowski and Klonowska (25), for instance, experimentally contrasted patients with bipolar disorder with healthy control participants to examine their potential differences in creativity as measured using the Revised Art Scale and the “inventiveness” subscale of the Berlin Intelligence Structure Test. The study looked for a potential association between creativity and mental illness and provided support for the madness-creativity nexus by showing higher scores among bipolar patients than among healthy persons on some creativity scales. Similarly, based on a cross-sectional design and a multimethod approach, Ruiter and Johnson (26) showed that mania risk is positively associated with self-reported creative achievement and creative personality. Johnson et al. (27) further confirmed the positive association between a validated measure of ambition and creativity.

However, after reviewing the previous literature, we found that there is a paradoxical or varied association between creativity and mental health. Relatively, the negative association between them seems more common than the positive association, with more evidence from the early literature, especially anecdotes, biographies, case studies and qualitative studies, pointing to a negative association between them [e.g., (18, 28)]. However, several humanistic and positive psychologists have argued that outstanding creativity constitutes a sure sign of better mental health (29, 30), a theory that is particularly supported by research using experimental, psychometric, psychiatric, and historiometric methods (31). Empirically, an increasing number of studies have provided evidence that simply engaging in creative activity can benefit physical and mental health [e.g., (32)]. For example, Shen et al. (33) reported a positive correlation between well-being and creativity and a partial mediating role of mindfulness in this positive association. Based on two meta-analyses, Yu and Zhang (34) revealed a negative association between creativity and negative well-being and a positive association between creativity and positive well-being. Further, Acar et al. (35) assessed the association between creativity and mental health or well-being by synthesizing 189 effect sizes obtained from 26 different studies and replicated a significantly positive, yet modest, link between creativity and mental health, implying that creative individuals tend to have higher well-being or that those with higher well-being tend to be more creative.

Moreover, several recent meta-analytical studies have provided direct evidence for the mad genius paradox, supporting a dichotomous association between creativity and mental health. That is, the answer to the question of whether creativity and mental health are positively or negatively correlated is that they correlate in both ways. For example, Taylor (36) conducted a systematic meta-analysis, wherein the link between mood disorder and creativity was evaluated using three separate approaches to determine whether creative persons are more likely to exhibit mood disorders, whether individuals with mood disorders behave more creatively, and whether a correlation exists between creativity and mood disorders as continuous constructs. The results across the three analyses varied. Simply put, creative (as opposed to non-creative) individuals indeed exhibited greater levels of mood disorders, which was true for all types of mood disorders except dysthymic disorder, while individuals with mood disorders did not exhibit different creativity levels than healthy controls. Although all mood disorder types were positively associated with creativity, there was a significantly stronger association with bipolar (and unspecified) than with unipolar disorder. Importantly, this correlation worked only when creativity was measured in terms of creative accomplishment and behavior. Differentiating between approach-based psychopathology (e.g., positive schizotypy) and avoidance-based psychopathology (e.g., anxiety), Baas et al. (37) conducted a meta-analysis of 57 empirical studies to determine possible linkages between risk of psychopathology and creativity in non-clinical samples and observed some meaningful results: a small positive relationship between positive schizotypy and creativity, a small negative correlation between negative schizotypy or anxiety and creativity, and the finding that the risk of bipolar disorder (e.g., hypomania) is positively associated with creativity, while depressive mood is negatively associated (albeit weakly) with creativity.

Ultimately, the pattern of association between creativity and mental health is complex. Early research, particularly case and clinical studies, tended to support the mad genius hypothesis, that is, either that mentally unhealthy people are more creative or that most creative people are mentally unhealthy. However, the results of a growing number of experimental and relatively tightly controlled clinical and subclinical studies have been mixed on this matter, and a notable portion of the research evidence does not fully support the traditional mad genius hypothesis. Thus, in response to these findings, several researchers have drawn on big data and large sample meta-analysis techniques, presenting generally contradictory results with both significant negative and significant positive associations between creativity and psychological well-being as well as with patterns of association not identical across studies or across measures.

## A New Theoretical Account Drawn From the Dual-Pathway Model

In this section, we conduct a critical analysis on the matter and now present our view. In addition to some potential confounding factors or mediation variables mentioned in previous studies [e.g., (38, 39)], we contend that the association between creativity and mental health varies according to whether creativity is considered a “disposition (body)” or a “strategy use.” Additionally, creativity is often negatively associated with mental health when it is measured as a relatively stable trait or disposition (personality/ability/achievement), i.e., a trait or ability/achievement, thus reflecting the persistence of creativity mentioned in the dual-pathway model. However, an association in the opposite direction is documented when creativity is considered a “use” or “technique/method,” i.e., a flexible approach mentioned in the proposed model or a strategy use. That is, when creativity is measured in terms of strategy use or situational variables, the association between creativity and mental health is mostly positive. We extend this theoretical idea more specifically below.

Nijstad et al. (40) proposed a new theory, namely, the dual-pathway model of creativity, which assumes that creativity is a function of cognitive flexibility and cognitive persistence and that dispositional or situational variables can influence creativity through their effects on flexibility, persistence, or both. After a careful review of previous studies, we find that most studies that took the approach of individual difference reported a negative association between creativity and mental health and tended to consider creativity as a type of dispositional or stable difference, that is, either a personality trait or a kind of capability. In fact, creativity is also considered a strategy, which is typically reflected in the expression of creative problem solving. Certain complex problems involve non-routine challenges with no immediately obvious solutions and are not solved until a creative strategy or a novel approach is used. That is, creative problem solving is a strategy or method that attempts to approach a correct solution or a challenge in an innovative way. Another line of studies is improving individuals' creativity performance through priming a creative mindset [e.g., (41)] or instructing them to think differently [e.g., (42)]. Accordingly, creativity is a level of flexibility in certain situations and can vary according to whether it is treated as a strategy. In this regard, creativity can benefit mental health and help individuals improve their mental health. There is no shortage of examples of creativity benefitting mental health in everyday life, such as through creative writing or creative language comprehension, such as humor understanding. For example, for the picture of the vomit in the toilet, creative cognitive reappraisal interprets it as she being inwardly happy that she finally had a child of her own, despite the fact that she threw up a lot. In general, people often experience a surge in emotion and happiness as a result of creative language use or appreciation (e.g., appreciating a visual metaphor) or of the resolution of interpersonal/social dilemmas by engaging in creative self-deprecation [e.g., disparagement humor; for a review, see (43)]. After reviewing previous studies, Table 1 was established to selectively list existing studies on the dichotomous association between creativity and mental health [we provide only a small number of studies supporting the positive association between creativity and mental illness; for more studies, please see some influential reviews or meta-analyses, e.g., (35, 36, 60)]. Specifically, Yu et al. (52), for instance, provided participants with descriptions of various scenarios that included some sort of mental distress and asked them to offer a resolution using one of three solution types: creative, literal, or problem restatement. The authors observed that the emotional positivity and strategic adaptability scores in metaphorical and literal solutions were significantly higher than those in problem restatement solutions. Additionally, the results showed that, compared with literal or problem restatement solutions, creative or metaphorical solutions activated two brain networks, each individually associated with basic metaphorical language processing and insightful problem solving, indicating that the use of creative solutions to resolve problems related to mental distress reliably prompts neural activities that generate positive effects (61). In a recent study by Wu et al. (49), creative reappraisal was reported to have superior performance in regulating emotion, especially negative emotion, accompanying the activation of a similar insight-related network encompassing the hippocampus, amygdala, and striatum. Theoretically, as mentioned above, emotional health is a key part of mental health that typically manifests in appropriately managing or regulating both positive and negative emotions. According to studies on dual-pathway models, positive and negative emotions can flexibly boost creativity or certain key processes of creativity, with negative moods being positively associated with cognitive persistence and positive activating moods being predominantly associated with stronger cognitive flexibility (62). This in turn implies that creativity can boost emotional health through either of these two opposing pathways.

**Table 1:** Example studies supporting the dichotomous link between creativity and mental health.

**No**.	**Sources**	**Approaches**	**Operational definition of creativity**	**Operational definition of well-being or mental health**	**Main findings**
1	Shen et al. (33)	Creativity as a strategy	Solving remote associate problems	Well-being was measured using the psychological well-being scale	Showing a positive effect of creativity on well-being
2	Conner et al. (44)	Creativity as a strategy	Creative activity was measured in the daily diary	Well-being was assessed through an eight-item Flourishing Scale.	Everyday creativity as a means of cultivating positive psychological functioning.
3	Bujacz et al. (45)	Creativity as a strategy	Three creative tasks: (43) invent titles for a cartoon, (1) list different uses for a rubber band, or (2) improve the design of a table for individuals with impaired vision.	Well-being was measured through a three-item positive emotion scale, a two-item autonomy scale and a two-item task absorption scale.	Engagement in creative tasks promoted autonomous self-expression and brought more positive emotions than noncreative ones.
4	DirŽyte et al. (46)	Creativity as a strategy	A five-item self-reported creativity questionnaire and a scale on the attitude to creativity modified from the creative mindsets scale.	Well-being was measured using an eight-item Flourishing Scale, which includes the dimensions of relationships, self-esteem, purpose, and optimism	Self-reported creativity and attitudes to creativity are significant, positive predictors of flourishing.
5	Fink et al. (47)	Creativity as a strategy	Participants were required to generate as many and as different ways as possible to reappraise presented anger-eliciting situations in a manner that reduces their anger. Neural activity elicited by the generation task was compared with the activity by the alternative uses task.	Well-being was measured by the reduction of anger intensity.	Creative reappraisal is an effective strategy to regulate an ongoing negative emotional state.
6	Wu et al. (48)	Creativity as a strategy	A humorous reappraisal (a creative way) is compared to an ordinary reappraisal.	Well-being was measured by the reduction of negative emotion intensity induced by negative pictures.	Humorous reappraisal was more effective in downregulating negative emotions and upregulating positive emotions both in the short and long term, altogether with the brain-related activation of creative/insightful restructuring and insight experience.
7	Wu et al. (49)	Creativity as a strategy	A series of generated creative reappraisals for standardized negative pictures were rated and provided to participants. There were two control conditions: an ordinary reappraisal condition and an objective description condition.	Well-being was measured by the reduction of negative emotion intensity induced by negative pictures.	Creative reappraisal had a long-lasting effect in reducing negative affect, which also makes standardized negative pictures have a positive rating in emotion.
8	Rominger et al. (50)	Creativity as a strategy	Creatively generating positive reappraisals of adverse events. Meanwhile, a problem-oriented generation task and a de-emphasizing task were used as controls.	Well-being was measured by the reduction of anxiety and anger intensity induced by adverse events.	Creative reappraisal is a useful strategy in regulating the negative experience elicited by adverse events.
9	Wu et al. (51)	Creativity as a strategy	Participants were requested to generate reappraisals of negative stimuli and then evaluate the creativity (rated by experts)	Well-being was measured by the reduction of anxiety and anger intensity induced by adverse events.	Individual creativity and reappraisal appropriateness were significant predictors of the regulating effects of the reappraisal for negative pictures and that creativity was the most dominant predictor.
10	Yu et al. (52)	Creativity as a strategy	The metaphorical (high creativity rating) solutions to mental distress problems were compared with literal solutions or problem-restatement solutions.	There was no significant difference in the emotional valence ratings between the metaphorical and literal solutions, but they were significantly higher than the problem-restatement solutions	The metaphorical solution to mental distress problem is a highly creative and useful strategy in regulating negative emotion and improving mental health.
11	Hu et al. (53)	Creativity as a strategy	A metaphorical restructuring intervention is viewed as creative as opposed to a literal restructuring intervention or a no restructuring problem restating intervention.	Well-being is reflected in the alleviation effectiveness of mental distress problems.	The mental distress of the metaphorical restructuring group significantly decreased after the intervention. Furthermore, this group had greater insightfulness during the intervention, and this insightfulness could predict the reduction of negative affect after the intervention.
12	Tan et al. (54)	Creativity as a strategy	Creativity was measured by the participants' self-report (study 1) or receiving a creativity priming task and executing an alternative use task (study 2).	Self-reported subjective well-being was measured by the Scale of Positive and Negative Experience and Satisfaction with Life Scale.	The study shows a positive, cross-sectional relationship between creativity and subjective well-being after controlling the effect of self-perceived stress and demographics. After controlling the effect of self-perceived stress, individuals receiving the creativity priming reported higher subjective well-being scores than their counterparts.
13	Miller et al. (55)	Creativity as a disposition	Individuals' self-rated creativity was measured through the 21-item Creativity Domain Questionnaire-Revised.	Well-being is assessed using a self-report measures of depression and hypo/mania over the past week from 397 participants previously diagnosed with BD.	Those self-reporting clinically significant depressive symptoms had significantly lower creativity scores (particularly in the domains of the drama, interaction and math/science) than those in the hypo/mania and no current symptom groups.
14	Gostoli et al. (56)	Creativity as a disposition	Creativity Assessment Packet encompassing divergent thinking test and creative personality test were used to measure creativity.	The 84-item psychological well-being questionnaire was used to measure well-being, altogether with the Temperament Evaluation of the Memphis, Pisa, Paris and San Diego-Auto questionnaire to assess subclinical psychopathological symptoms.	Significant positive correlations between creativity and bipolar disorder vulnerability, especially hyperthymia, were observed. Creativity was poorly linked to psychological well-being subscales, except autonomy and personal growth.
15	Johnson et al. (27)	Creativity as a disposition	Creativity was determined through multiple measures, including the unusual use test (uniqueness), subjective creativity evaluation, and creative achievement questionnaire.	Well-being was also measured through multiple instruments, including the Beck Depression Inventory, Altman Self-Rating Mania Scale, Modified Hamilton Rating Scale for Depression, and the Young Mania Rating Scale.	Persons with bipolar disorder demonstrate significantly more heterogeneity in creative accomplishment levels compared with those with no bipolar disorder.
16	McNeil and Clinic (57)	Creativity as a disposition	Creative ability was determined through the independent ratings of each participant's activities and accomplishments, together with the information on the questionnaires and from the creative product.	Well-being was mainly determined based on participants' records of the Btspebferg Hospital Psychiatric, Military Service, and Psychiatric Register of the Human Genetics Institute.	There is a significant, positive association between creative ability and mental illness.
17	Rybakowski and Klonowska (25)	Creativity as a disposition	The Revised Art Scale and the “inventiveness” part of the Berlin Intelligence Structure Test were adopted to measure creativity.	The Oxford-Liverpool Inventory of Feelings and Experiences was used to estimate schizotypal. Well-being was established based on the aforementioned assessment and the medical diagnosis of bipolar disorder.	The bipolar patients obtained significantly higher scores on the BIS-total as well as on the verbal part of the test, showing higher scores on some creativity scales in bipolar patients compared with the healthy.
18	Santosa et al. (58)	Creativity as a disposition	Creativity was measured through four instruments: the Barron–Welsh Art Scale (BWAS-Total, and two subscales, BWAS-Dislike and BWAS-Like), the Adjective Check List Creative Personality Scale (ACL-CPS), and the Torrance Tests of Creative Thinking – Figural (TTCT-F) and Verbal (TTCT-V) versions	Well-being was determined by whether the participant had suffered from euthymic bipolar (BP) or unipolar major depressive disorder (MDD).	Results showed BP and creative discipline controls (CC) compared to healthy controls scored significantly higher on BWAS-Total and BWAS-Dislike. The CC compared to MDD scored significantly higher on TTCT-F.
19	Taylor et al. (59)	Creativity as a disposition	Creativity was measured through self-reported engagement in productive creative activity.	Well-being was determined based on multiple criteria: persons who had received a clinical diagnosis of self-reporting bipolar disorder, confirmed by the Mood Disorders Questionnaire based on DSM-IV diagnostic criteria for BD	There is a positive association between creativity and bipolar disorder.

Overall, the present research provides useful insights into the association between creativity and mental health, which could explain some of the complex cognitive and neural processes involved in both creativity and psychopathology and has the potential to paint a clearer picture of some overlapping mechanisms in both constructs rather than linking creativity generally to “madness.”

## Discussion and Conclusion

In this study, we review previous research on the association between mental health and creativity. Overall, a dichotomous association is documented, namely, both positive and negative associations are observed. Based on previous findings and the dual-pathway model of creativity, we offer a new view, wherein the positive–negative nature of the association between creativity and mental health is largely determined by the nature of creativity and/or its corresponding measurements. When creativity is conceptualized or operationalized as dispositional, the association is negative, whereas it is positive when creativity is treated as a strategy (e.g., as an intervention method or regulation activity). Indeed, some studies have provided direct support for this idea. For example, Acar et al. (35) found that the approaches to measure creativity are what account for the variation in the association, with a stronger association occurring when creativity is measured by instruments focusing on creative activity and behavior than by those looking at divergent thinking tasks. Nevertheless, we also acknowledge that some alternative explanations cannot be excluded without rigorously controlled studies. For example, Paek et al. (63) conducted a meta-analysis using 89 studies to examine the overall relationships between the most common psychopathologies and little-c creativity and revealed that the overall mean effect size was not different from zero but varied, with effect sizes ranging from −0.97 to 0.95, and 54% of the total effect sizes being below zero and 44.4% of the total effect sizes being above zero. These results actually confirmed the paradoxical association between creativity and mental health. Additionally, their moderator analyses showed that effect sizes varied by the assessment of both psychopathology and creativity as well as by level of intelligence. Furthermore, Drapeau and DeBrule (64) revealed the role of moderating variables, such as the assessment or domain of creativity, in the association between creativity and mental health, wherein the relationships among hypomania, creativity (divergent thinking and creative achievement), and suicidal ideation in college students were examined. Their results showed that, among the creative domains surveyed, students with high creative achievement in architectural design may experience the highest risk for significant suicidal ideation; furthermore, students with visual arts, creative writing, theater/film, and dance achievements were at moderate risk for significant suicidal ideation, thus implying that the (negative) association between creativity and mental health may vary according to the domain of creativity. Future studies could systemically re-examine this new account and exclude alternative accounts [e.g., the inverted-U relationship between creativity and mental illness, see (11); the shared vulnerability model; see Carson (65)] using additional empirical investigations. For example, Ghadirian et al. (66) reported that creativity was at its highest level in patients who suffered a moderate level of manic-depressive illness, whereas the lowest creativity score appeared in the group of patients identified as severely ill.

Taken together, this research provides a novel and useful perspective to evaluate the relationship between creativity and mental health, which has many theoretical or practical implications. Specifically, this research, as a perspective study or mini review, focuses on the association between creativity and mental health, attempting to reconcile the diverging and even contradictory empirical findings regarding the relationship between creativity and mental health. On the one hand, the present research contributes greatly to facilitating positive mental health by showing the positive role of creativity as a strategy to regulate negative emotion and improve positive mental health by creatively reducing negative experiences and insightful or creative reappraisal toward negative situations or things. On the other hand, the current study evaluates a longstanding controversy within the domain of mental health and/or creativity. This research has important implications for future studies on the creativity-well-being nexus and health practices. First, this study hints that, when future research attempts to investigate the relationship between mental health and creativity, they should distinguish between individuals' dispositional creativity and strategic creativity and develop creativity-based mental health adjustment strategies and skills to improve mental health literacy and coping skills. Another important aspect of this study was to deepen the analysis of the concept and structure of mental health literacy and to focus on exploring the constructs, skills, and mechanisms associated with creativity. Practically, this research will help to properly understand the theoretical relevance of creativity and mental health and its models and nudge the application of creativity in the mental health field. In this case, special focus is given to the impact of cognitive reappraisal of creativity and emotional cognitive reappraisal on (emotional) mental health, considering the development of creative regulation strategies and/or creative reappraisal skills as an important component of mental health literacy.

## Author Contributions

RZ and WS conceptualized and designed the work. RZ, ZT, and WS drafted the manuscript, with critical comments from QX and FL. All authors contributed to the article and approved the submitted version.

## Funding

This work was supported by the research fund of Jiangsu Provincial Key Constructive Laboratory for Big Data of Psychology and Cognitive Science (No. 72592162002G), the Guangdong Education Science 2021 Education Science Planning Project A study on digital mental health evaluation system for college students (No. 2021GXJK287) and Guangdong Social Science Planning 2021 project Construction of the Value-added Evaluation system of Adolescents' Mental Health based on Digitization (No. GD21CJY12).

## Conflict of Interest

The authors declare that the research was conducted in the absence of any commercial or financial relationships that could be construed as a potential conflict of interest.

## Publisher's Note

All claims expressed in this article are solely those of the authors and do not necessarily represent those of their affiliated organizations, or those of the publisher, the editors and the reviewers. Any product that may be evaluated in this article, or claim that may be made by its manufacturer, is not guaranteed or endorsed by the publisher.
